# Prenatal findings in a fetus with X-linked recessive type of chondrodysplasia punctata (CDPX1): a case report with novel mutation

**DOI:** 10.1186/s12887-019-1629-x

**Published:** 2019-07-23

**Authors:** Guannan He, Yan Yin, Jing Zhao, Xueyan Wang, Jiaxiang Yang, Xi Chen, Li Ding, Yan Bai

**Affiliations:** 1 Department of Ultrasound, Women and Children’s Hospital of Sichuan Province, No.290, Shayan West 2nd Road, Chengdu, 610031 Sichuan Province China; 2 Department of Prenatal Diagnosis, Women and Children’s Hospital of Sichuan Province, No.290, Shayan West 2nd Road, Chengdu, 610031 Sichuan Province China; 3 Department of Radiology, Women and Children’s Hospital of Sichuan Province, Chengdu, 610031 China

**Keywords:** X-linked recessive chondrodysplasia punctate, Arylsulfatase E, Prenatal ultrasound, Whole exome sequencing

## Abstract

**Background:**

X-linked recessive chondrodysplasia punctate (CDPX1) is a rare congenital disorder of bone and cartilage development, caused by a mutation in the arylsulfatase E (ARSE) gene located on chromosome Xp22.3. Although most of the affected men had mild symptoms, some had more severe symptoms, and had a poor prognosis.

**Case presentation:**

We present the case of a male fetus diagnosed with CDPX1. Ultrasound clearly showed that hypoplasia of the midface, flatness of face, low flatness of the nose, collapse of the tip of the nose, accompanied by severe spinal stenosis and secondary ossification center of the femoral metaphysis appeared in advance. Chromosome analysis of the amniotic fluid cells revealed 46, XY. Whole exome sequencing showed that there was a novel missense mutation of c.640G > A in ARSE gene on X chromosome. Three protein function prediction software FATHMM、Polyphen-2、PROVEAN have shown that the novel missense mutation of c.640G > A in this study was pathogenic.

**Conclusions:**

Our case is a novel mutation and presents a typical characterization of the disease, which can expand the spectrum of mutations of the ARSE gene and is helpful for prenatal ultrasound diagnosis of this disease.

## Background

X-linked recessive chondrodysplasia punctate (CDPX1; MIM#302950) is a rare congenital disorder of bone and cartilage development, caused by a mutation in the arylsulfatase E (*ARSE*, MIM*300180) gene located on chromosome Xp22.3 [1]. It was first described by Curry et al. [2], and the incidence of CDPX1 is 1:100000 live births [3]. Maternal immune system disease, exposure to alcohol, or drugs was aetiological factor of chondrodysplasia punctate (CDP) [4–6]. CDPX1 is characterized by punctate calcification in areas of endochondral bone formation, leading to stippled epiphyses, severe nasal and midfacial hypoplasia; ectopic calcifcations often affecting the airways and in particular the tracheal cartilage; and distal phalangeal hypoplasia [7]. However, the condition of patient is only noticed after birth because of facial features or breathing problems. There have been few reports on prenatal diagnosis of CDPX1 by ultrasound examination [8, 9]. Here we report a fetus with CDPX1, who was affected by a novel ARSE gene mutation.

## Case presentation

A 25-year-old woman, gravida 1 para 0, was referred to our hospital at 23 weeks of gestation because of ultrasound abnormality of the fetus that abnormal curvature of spine. Pregnant denied history of immune system disease, exposure to alcohol or drugs such as warfarin and phenytoin.

**Fig. 1:**
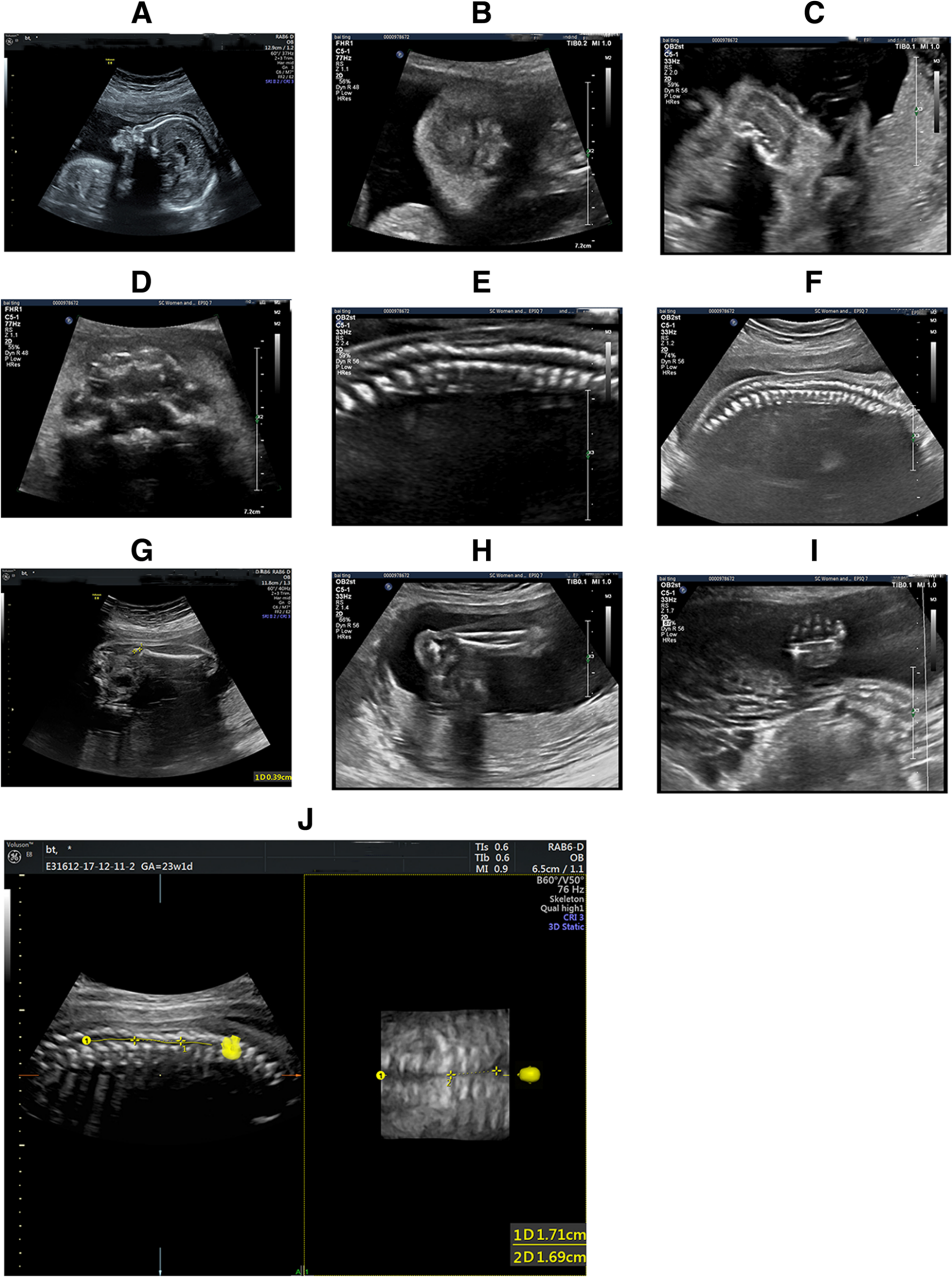
Antenatal ultrasonography features in our patient. **a** & **b**, Sagittal and coronal views of the fetal face showing facial flat, a depressed nose bridge, and collapsed nasal tip. **c**. Horizontal cross view of the fetal nasal tip show depressed, collapsed, and flat of the nasal tip. **d**. Transverse view of the upper alveolus shows that the upper alveolus is square. **e** & **f**. Sagittal view of the fetal spine shows changes in physiological curvature, T_9_-L_1_ spinal canal is narrow, the diameter of the thoracic spinal canal above the stenosis segment was about 4.1 mm, and that of the lumbar segment below the stenosis segment was about 4.0 mm. **j**. Three-dimensional ultrasound imaging of the fetal spine revealed T_9_-L_1_ spinal canal is narrow. **g**. Long axial view of the fetal femur show that early secondary ossification centers. **h** & **i**. There was no obvious abnormality of fetal ankle joint and hand ossification

Ultrasound examination with 5-7 M Hz probe of the fetal system was then performed. Prenatal ultrasound phenotype are as followed: Fetal external genitals is male, face is flat, nose bridge is depressed, nasal tip is collapse (Fig. 1a-c), and the upper alveolar is square (Fig. 1d). The spine loses normal physiological curvature, some vertebral bodies are small, and the spinal canal is narrow. The T9-L1vertebra is most obvious (Fig. 1e, f and j). The inner diameter of the narrowest part is only 2 mm. The diameter of the thoracic spinal canal above the stenosis segment was about 4.1 mm, and that of the lumbar segment below the stenosis segment was about 4.0 mm. Secondary ossification centers appear in the epiphyses of both femurs (Fig. 1g, h, i). Based on these ultrasound phenotypes, we recommend that the patient undergo fetal magnetic resonance and amniotic fluid testing to further clarify the diagnosis.

**Fig. 2:**
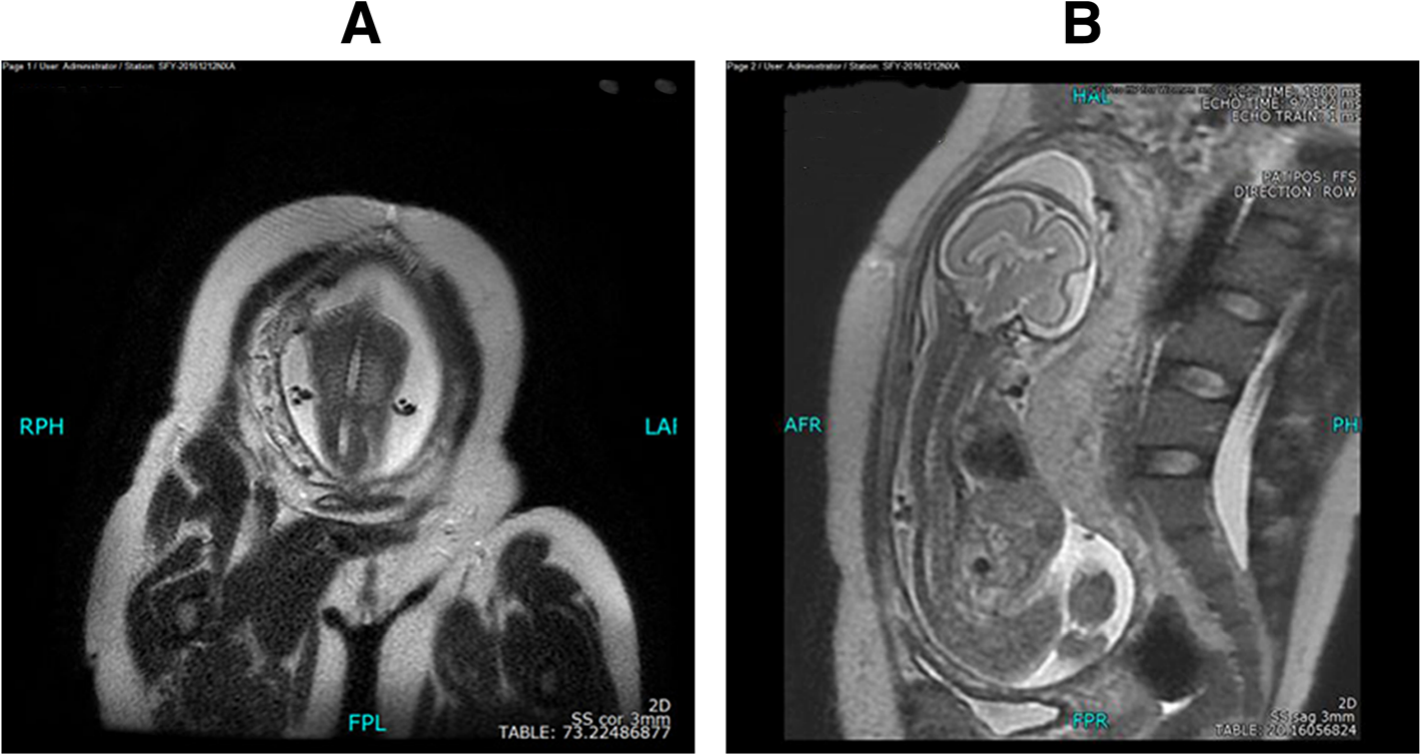
MRI features in our patient. **a** & **b**. MRI showed the disappeared normal curvature of the fetal spine, partial depression of lower thoracic segment, and abnormal and narrow down of the spinal canal

The GE 1.5 T MRI showed the disappeared normal curvature of the fetal spine, partial depression of lower thoracic segment, thinner of muscular layer, and abnormal and narrow down of the spinal canal in the range of about 1.9 cm. Axial view shows abnormal shape of spinal canal and thinning of adjacent spinal cord (Fig. 2).

### Genetic analysis

**Fig. 3:**
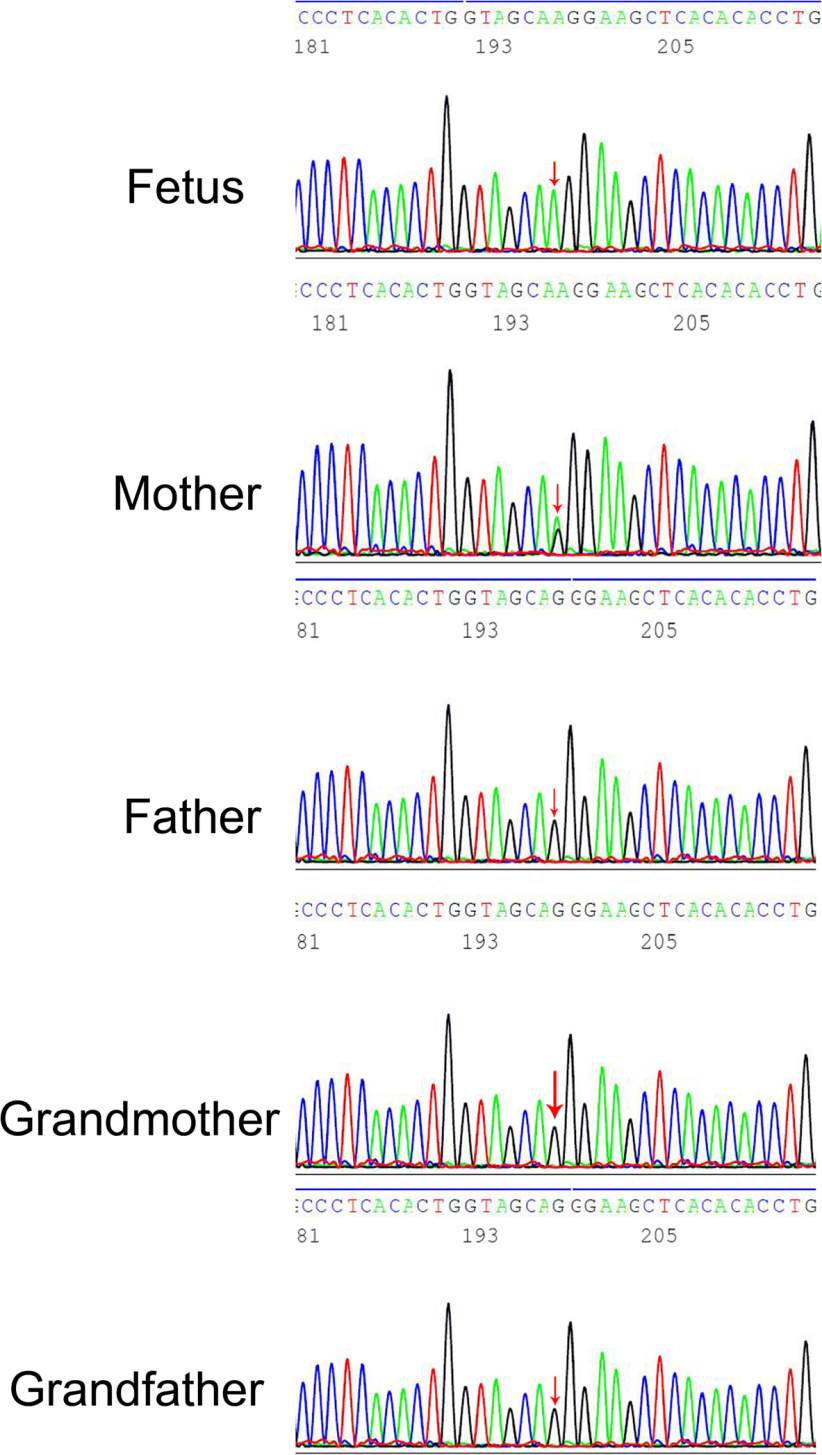
The sequencing results of the TNSALP gene in pedigree

Chromosome analysis of the amniotic fluid cells was performed with conventional G-banding and revealed 46, XY without any structural aberrations. Parents were informed of the purpose of the study and signed the informed consent. Genomic DNA was isolated from amniotic fluid and peripheral blood leukocytes using the DNeasy Blood & Tissue Kit (Dusseldorf, Germany) and QIAamp DNA Blood Mini Kit (Dusseldorf, Germany) according to the manufacturer’s instructions. Whole exome sequencing: Roche SeqCap EZ MedExome Enrichment Kit was used to perform exome target enrichment. The captured library was sequenced on the illumina Hiseq X-ten Sequencer with PE150 and mean sequencing depth of 100×, and more than 95% of the area with a coverage of more than 20×. Raw data of exome sequencing in FASTQ format and processed with bioinformatization. A novel missense mutation of ARSE gene c.640G > A (p.G214R) on X chromosome was found in fetus (refer to the human genome hg19 reference sequence in UCSC database). To further confirm the variant, Sanger sequencing was performed in pedigree. The mother carried the mutation, the maternal grandparents did not have the mutation, the fetus inherited the mutation from the mother (Fig. 3). Three protein function prediction software FATHMM、Polyphen-2、PROVEAN have shown that the novel missense mutation of c.640G > A in this study was pathogenic.

## Discussion and conclusions

CDP is a skeletal disorder characterized by premature ossification of cartilage, which contain different subtypes, including rhizomelic CDP (type1, 2 and 3), CDPX1, X-linked dominant chondrodysplasia punctate (CDPX2) [10]. CDP is commonly seen in the epiphyses of long bones, the spine, and other uncalcified cartilage, as well as in the trachea and the ends of the ribs.

In previous report, CDPX1 is characterized by brachytelephalangy, punctate calcifications affecting the vertebrae, carpal and metacarpal bones, maxillonasal hypoplasia, and mild developmental delay [11]. Punctate calcification is not a specific feature of the disease and has been found in many diseases, such as Zellweger syndrome, Warfarin embryopathy, Vitamin K reductase deficiency, Trisomy 21, Trisomy 18, and Smith-Lemli-Opitz syndrome etc. [1]. Calcifications can be detected by prenatal ultrasound and x-rays. With the increase of gestational weeks, cartilage begins to ossify and the lesion is often covered by normal calcification, which brings trouble to prenatal diagnosis. Another major abnormality is vertebral involvement, including poor ossification, abnormal shape (wedge, cone, flat), and accompanied by changes in the physiological curvature of the spine, which can reflect the degree of vertebral involvement by observing the physiological curvature. The most common facial findings include midface dysplasia, nasal dysplasia and flat cheekbones, similar to prenatal exposure to warfarin in fetuses. Most fetuses are associated with intrauterine growth restriction. There have been few reports on prenatal diagnosis of CDPX1 by ultrasound examination. Symptoms of CDPX1 fetus diagnosed by prenatal ultrasound include nasal hypoplasia, hydramnios, oligohydramnios, long bone dysplasias and vertebrae abnormalities [8, 9, 12]. Although nasal hypoplasia and long bone dysplasias are often considered characteristic changes of Trisomy 21, chromosomal abnormalities can be excluded by amniotic fluid karyotype analysis. Compared with other types, CDPX1 has mild clinical manifestations and good prognosis. Several cases of CDPX1 with disc dislocation or spinal stenosis have been reported with poor prognosis [13, 14].

In our patient, the clinical diagnosis of CDPX1 was based on the ultrasound examination findings with typical facial hypoplasia, vertebrae abnormalities, and premature ossification of the metaphysis of the diaphysis no maternal history of use of warfarin or phenytoin, an inheritance inheritance pattern consistent with X-linked recessive inheritance, and male sex. CDPX1 is caused by a mutation in the gene, which encodes the Golgi enzyme ARSE, antibodies to its enzymes can cross the placenta to the fetus. Genetic analysis revealed that a novel missense mutation of ARSE gene c.640G > A on X chromosome was found in fetus in this report. Further family study revealed that the fetus inherited the mutation from the mother of the novel mutation.

In conclusion, we report a case of a fetus with CDPX1 in whom a novel missense mutation was identified. Our patient was diagnosed by prenatal ultrasound, MRI and genetic analysis. This case is a novel mutation and presents a typical characterization of the disease, which can expand the spectrum of mutations of the ARSE gene and is helpful for prenatal ultrasound diagnosis of this disease.

## Data Availability

All data generated or analyzed during this study are included in this published article.

## Abbreviation

*ARSE*: arylsulfatase E
CDP: Chondrodysplasia punctate
CDPX1: X-linked recessive chondrodysplasia punctate
CDPX2: X-linked dominant chondrodysplasia punctate
CMV: cytomegalovirus
